# The MAO Inhibitor Tranylcypromine Alters LPS- and Aβ-Mediated Neuroinflammatory Responses in Wild-type Mice and a Mouse Model of AD

**DOI:** 10.3390/cells9091982

**Published:** 2020-08-28

**Authors:** HyunHee Park, Kyung-Min Han, Hyongjun Jeon, Ji-Soo Lee, Hyunju Lee, Seong Gak Jeon, Jin-Hee Park, Yu Gyung Kim, Yuxi Lin, Young-Ho Lee, Yun Ha Jeong, Hyang-Sook Hoe

**Affiliations:** 1 Department of Neural Development and Disease, Korea Brain Research Institute (KBRI), 61, Cheomdan-ro, Dong-gu, Daegu 41062, Korea; hyunhee16hh@gmail.com (H.P.); hkm5344@kbri.re.kr (K.-M.H.); newace16@kbri.re.kr (H.J.); su943c@kbri.re.kr (J.-S.L.); hjlee@kbri.re.kr (H.L.); jsg7394@kbri.re.kr (S.G.J.); Mingmeng1005@gmail.com (J.-H.P.); cosmos0468@gmail.com (Y.G.K.); 2 Department of Brain and Cognitive Sciences, Daegu Gyeongbuk Institute of Science & Technology, Daegu 42988, Korea; 3 Department of Pharmacology, School of Dentistry, Kyungpook National University, Daegu 41940, Korea; 4 Research Center of Bioconvergence Analysis, Korea Basic Science Institute (KBSI), Ochang, Cheongju, Chungbuk 28119, Korea; linyuxi@kbsi.re.kr (Y.L.); mr0505@kbsi.re.kr (Y.-H.L.); 5 Neurovascular Research Group, Korea Brain Research Institute (KBRI), 61, Cheomdan-ro, Dong-gu, Daegu 41062, Korea; 6 Bio-Analytical Science, University of Science and Technology (UST), Gajeong-ro, Yuseong-gu, Daejeon 34113, Korea

**Keywords:** neuroinflammation, MAO inhibitor, microglia, amyloid beta, LPS

## Abstract

Monoamine oxidase (MAO) has been implicated in neuroinflammation, and therapies targeting MAO are of interest for neurodegenerative diseases. The small-molecule drug tranylcypromine, an inhibitor of MAO, is currently used as an antidepressant and in the treatment of cancer. However, whether tranylcypromine can regulate LPS- and/or Aβ-induced neuroinflammation in the brain has not been well-studied. In the present study, we found that tranylcypromine selectively altered LPS-induced proinflammatory cytokine levels in BV2 microglial cells but not primary astrocytes. In addition, tranylcypromine modulated LPS-mediated TLR4/ERK/STAT3 signaling to alter neuroinflammatory responses in BV2 microglial cells. Importantly, tranylcypromine significantly reduced microglial activation as well as proinflammatory cytokine levels in LPS-injected wild-type mice. Moreover, injection of tranylcypromine in 5xFAD mice (a mouse model of AD) significantly decreased microglial activation but had smaller effects on astrocyte activation. Taken together, our results suggest that tranylcypromine can suppress LPS- and Aβ-induced neuroinflammatory responses in vitro and in vivo.

## 1. Introduction

Alzheimer’s disease (AD) is a neurodegenerative disease associated with amyloid β (Aβ) and Tau pathologies, for which treatment options remain limited [1,2]. Interestingly, several recent studies have shown that neuroinflammation causes synaptic impairment and cognitive dysfunction, indicating that neuroinflammation is directly and/or indirectly involved in neurodegenerative diseases [3]. Aβ plaques induce neuroinflammatory responses by activating microglial and astrocyte activation in AD patients and mouse models of AD [4]. In particular, Aβ oligomers or fibrils interact with Toll-like receptors (TLRs; TLR4, TLR6, TLR9, and TLR2) expressed on microglial cells to promote the release of proinflammatory cytokines, including COX-2, IL-1β, and IL-6 [5]. These TLRs also bind to lipopolysaccharide (LPS), leading to activation of proinflammatory cytokine genes via MyD88-dependent or MyD88-independent pathways to induce inflammatory responses [6]. Thus, anti-inflammatory drugs may be useful to prevent/treat both neuroinflammation-related and neurogenerative diseases (e.g., AD).

Several studies have demonstrated that monoamine oxidase (MAO) is involved in neuroinflammation [7], triggering of apoptosis [8], failure of aggregated protein clearance [9], AD [8,10], Parkinson’s disease (PD) [11,12], and Lewy body diseases with dementia [13]. In addition, MAO activation leads to cognitive impairment [14], destroys cholinergic neurons, and causes disorders of the cholinergic system [15]. Activated MAO-B (monoamine oxidase-B) contributes to the formation of amyloid plaques and neurofibrillary tangles [16,17]. These observations suggest that regulating MAO activity might have therapeutic potential for neurodegenerative diseases (e.g., AD, PD).

Tranylcypromine (tranylcypromine hydrochloride; trans-2-phenylcyclopropanamine hydrochloride; TCP; Parnate; 2-PCPA) is a lysine-specific histone demethylase 1 (LSD1) inhibitor that also irreversibly blocks MAO-A (monoamine oxidase-A) and MAO-B [18,19]. Monoamine oxidase inhibitors (MAOIs) like tranylcypromine are well-known drugs that have been used clinically for the treatment of depression, anxiety, and PD [20]. As an LSD1 inhibitor, tranylcypromine has been tested for the treatment of cancer by regulating leukemia cell maturation [21]. A recent study demonstrated that tranylcypromine has an antiosteoporotic effect in an LPS-mediated osteolysis mouse model [22]. Another study found that tranylcypromine downregulates LPS-induced TNF-α, IL-1β, and IL-6 levels in a rat model of depression [23]. Moreover, tranylcypromine inhibits Aβ-induced toxicity in primary cortical neurons [24]. However, whether tranylcypromine can alter Aβ- and/or LPS-induced neuroinflammatory responses in the brain and its mechanisms of action remain to be determined.

Here, we investigated whether tranylcypromine affects LPS-induced neuroinflammatory responses and found that tranylcypromine-treated BV2 microglial cells selectively decreased LPS-induced proinflammatory cytokine levels. Tranylcypromine regulated TLR4/ERK/STAT3 signaling to reduce LPS-induced microglial proinflammatory cytokine levels in BV2 microglial cells. Importantly, tranylcypromine modulated LPS-induced microglial activation as well as proinflammatory cytokine COX-2 and IL-6 levels in wild-type mice. In addition, tranylcypromine modulated Aβ-induced microglial activation in 5xFAD mice but had smaller effects on astrocyte activation. Taken together, these results demonstrate that tranylcypromine affects LPS- and Aβ-induced neuroinflammatory responses in BV2 microglial cells, wild-type mice, and a mouse model of AD.

## 2. Materials and Methods

### 2.1. MAO Inhibitor Tranylcypromine

The MAO inhibitor tranylcypromine was purchased from Selleck Chemicals ((Figure 2F, Houston, TX, USA) and MedKoo Biosciences, Inc. (Morrisville, NC, USA).

### 2.2. Cell Lines

BV2 microglial cells (a generous gift from Dr. Kyung-Ho Suk) were used to assess the effects of tranylcypromine on LPS-induced neuroinflammatory responses and were maintained in high-glucose DMEM (Invitrogen, Carlsbad, CA, USA) with 5% fetal bovine serum (FBS, Invitrogen) in a 37 °C incubator with 5% CO_2_. To test whether tranylcypromine modulates LPS-induced neuroinflammation in primary astrocytes, mixed glial cultures were prepared as previously described [25].

### 2.3. Wild-Type Mice and 5xFAD Mice

The regulatory effects of tranylcypromine on neuroinflammatory responses in vivo were assessed in male C57BL6/N (wild-type) mice (Orient-Bio Company, Gyeonggi-do, Korea) and F1 generation 5xFAD mice (a mouse model of AD; stock# 34848-JAX, B6Cg-Tg APPSwFlLon, PSEN1*M146L*L286V6799Vas/Mmjax, Jackson Laboratory). All experiments were performed in accordance with approved animal protocols and guidelines established by the Korea Brain Research Institute (IACUC-19-00049, IACUC-19-00042). All mice were housed in a pathogen-free facility with 12 h of light and dark at a temperature of 22 °C.

Wild-type mice were intraperitoneally (i.p.) administered tranylcypromine (3 mg/kg) or PBS daily for 3 days. On day 3, the wild-type mice were injected with LPS (Sigma, *Escherichia coli,* 10 mg/kg, i.p.) or PBS. Eight hours after injection, the mice were sacrificed and fixed with 4% paraformaldehyde, and coronal slices of the brain with a thickness of 35 μm were obtained using a cryostat. The slices were then immunostained with anti-Iba-1 (1:500, Wako, Osaka, Japan), anti-GFAP (1:500, Neuromics, Edina, MN, USA), anti-IL-1β (1:200, Abcam, Cambridge, UK), anti-IL-6 (1:200, Santa Cruz Biotechnology, Santa Cruz, CA, USA), anti-CD11b (1:200 Abcam), anti-NeuN (1:500 Millipore, Middlesex, MA, USA), or anti-COX-2 (1:200, Abcam) antibodies.

To assess the effect of tranylcypromine on neuroinflammation in a mouse model of AD, male 5xFAD mice were injected with tranylcypromine (3 mg/kg, i.p.) or PBS daily for 1 week, and immunohistochemistry was performed with anti-Iba-1 or anti-GFAP antibodies. Image J software was used for semi-automated data analysis, and the results were confirmed by an independent researcher who did not participate in the current experiments.

### 2.4. MTT Assay

The 3-(4,5-dimethylthiazol-2-yl)-2,5-diphenyltetrazolium bromide (MTT) assay was used to assess the cytotoxic effects of tranylcypromine in BV2 microglial cells. After treatment with tranylcypromine (1, 5, 10, 25, or 50 μM) or vehicle (1% DMSO) for 24 h in the absence of FBS, cells seeded in 96-well plates were incubated with 0.5 mg/mL MTT at 37 °C and 5% CO_2_ for 3 h. Cytotoxicity was assessed by reading the absorbance at 570 nm using a microplate reader (SPECTROstar Nano, BMG Labtech, Germany).

### 2.5. Cell Counting Kit-8 (CCK-8) Assay

To measure the effects of post- or pretreatment with tranylcypromine on cell viability and cytotoxicity, we performed the Cell Counting Kit (CCK) assay (Dongin Biotech Co., Ltd., Seoul, Korea) according to the manufacturer’s instructions. To assess post treatment effects, BV2 microglial cells seeded in 96-well plates were treated with LPS (1 μg/mL) or PBS for 30 min, followed by treatment with tranylcypromine for 5.5 h. To assess pretreatment effects, BV2 microglial cells were treated with tranylcypromine or 1% DMSO for 30 min, followed by treatment with LPS for 5.5 h. In both cases, the final treatment was followed by incubation with 10% CCK solution in DMEM at 37 °C for 30 min in the dark. The absorbance was then read at 450 nm using a microplate reader (SPECTROstar Nano, BMG Labtech, Germany).

### 2.6. Reverse Transcription-Polymerase Chain Reaction (RT-PCR)

The ability of tranylcypromine to regulate proinflammatory cytokine levels induced by LPS was assessed in BV2 microglial cells. After pretreatment with tranylcypromine (5 μM) or vehicle (1% DMSO) for 30 min, the cells were treated with LPS (1 μg/mL) or PBS for 5.5 h. A Superscript cDNA Premix Kit II with oligo dT primers (GeNet Bio, Daejeon, Korea) was used to obtain cDNA by reverse transcription of total RNA extracted using TRIzol (Ambion, Life Technologies, Carlsbad, CA, USA). The cDNA was then used as the template in RT-PCR using Prime Taq Premix (GeNet Bio). RT-PCR was performed as previously described [25] using the following primers: IL-1β: forward (F)′, AGC TGG AGA GTG TGG ATC CC, and reverse (R)′, CCT GTC TTG GCC GAG GAC TA; IL-6: F′, CCA CTT CAC AAG TCG GAG GC, and R′, GGA GAG CAT TGG AAA TTG GGG T; IL-18: F′, TTT CTG GAC TCC TGC CTG CT, and R′, ATC GCA GCC ATT GTT CCT GG; COX-2: F′, GCC AGC AAA GCC TAG AGC AA, and R′, GCC TTC TGC AGT CCA GGT TC; iNOS: F′, CCG GCA AAC CCA AGG TCT AC, and R′, GCA TTT CGC TGT CTC CCC AA; GAPDH: F′, CAG GAG CGA GAC CCC ACT AA, and R′, ATC ACG CCA CAG CTT TCC AG. After separation by electrophoresis on 1.2% agarose gels with EcoDye (1:5000, Biofact, Daejeon, Korea), Fusion Capt Advance software (Vilber Lourmat, Eberhardzell, Germany) was used to analyze images of the RT-PCR products.

### 2.7. Real-Time PCR (Q-PCR)

To determine if tranylcypromine alters LPS-mediated anti-inflammatory cytokine levels, BV2 microglial cells were pretreated for 30 min with LPS (1 μg/mL) or PBS and then, treated with tranylcypromine (5 μM) or vehicle (1% DMSO) for 5.5 h. Total RNA extracted with TRIzol (Ambion) according to the manufacturer’s protocol was reverse transcribed into cDNA by using Superscript cDNA Premix Kit II with oligo dT primers (GeNet Bio). Fast SYBR Green Master Mix (Thermo Fisher Scientific, San Jose, CA, USA) and a QuantStudio 5 Real-Time PCR System (Thermo Fisher Scientific) were used for real-time PCR. Real-time PCR was performed as previously described [25] using the following primers: COX-2: Forward (F)′, CCA CTT CAA GGG AGT CTG GA, and Reverse (R)′, AGT CAT CTG CTA CGG GAG GA; IL-6: F′, CCA CGG CCT TCC CTA CTT C, and R′, TTG GGA GTG GTA TCC TCT GTG A; iNOS: F′, GGA TCT TCC CAG GCA ACC A, and R′, TCC ACA ACT CGC TCC AAG ATT; IL-1β: F′, TTG ACG GAC CCC AAA AGA TG, and R′, AGG ACA GCC CAG GTC AAA G; IL-10: F′, GAT GCC CCA GGC AGA GAA, and R′, CAC CCA GGG AAT TCA AAT GC; IL-4: F′, CCC ACC TGC TTC TCT GAC TAC A, and R′, CAG CGC TAT CCA GGA ACC A; GAPDH: F′, TGG GCT ACA CTG AGG ACC ACT, and R′, GGG AGT GTC TGT TGA AGT CG. The Ct value for *gapdh* was used to normalize the cycle threshold (Ct) values of anti-inflammatory cytokine mRNA.

### 2.8. Western Blotting

The effects of tranylcypromine on LPS-induced ERK signaling were assessed in BV2 microglial cells treated successively with LPS (1 μg/mL) or PBS for 45 min and tranylcypromine (5 μM) or vehicle (1% DMSO) for 45 min. The cells were then prepared for western blotting by lysis in Cell Lysis Buffer (ProPrep, iNtRON Biotechnology, Inc., Seongnam, Korea). After centrifuging the lysate at 12,000 rpm for 15 min, the supernatant was collected. The protein samples were separated by SDS gel electrophoresis and then, electrotransferred to a polyvinylidene difluoride (PVDF) membrane, which was blocked with 5% skim milk or 5% BSA and incubated with anti-ERK (1:1000, Santa Cruz Biotechnology) or anti-p-ERK (1:1000, Cell Signaling) antibodies overnight. After incubating the membranes with horseradish peroxidase-conjugated secondary antibody for 1 h, detection was achieved with ECL Western Blotting Detection Reagent (GE Healthcare, Chicago, IL, USA). Fusion Capt Advance software (Vilber Lourmat) was used to acquire and analyze images.

### 2.9. Enzyme-Linked Immunosorbent Assay (ELISA)

To assess whether tranylcypromine affects proinflammatory cytokine levels, BV2 microglial cells were treated with LPS (200 ng/mL) or PBS for 30 min, followed by treatment with tranylcypromine (5 μM) or vehicle (1% DMSO) for 23.5 h. Protein levels of the proinflammatory cytokines IL-6 and TNF-α in the conditioned media were measured using ELISA kits (Invitrogen, Cat. No. 88-7064-88 and 88-7324-88, respectively) in accordance with the manufacturer’s instructions.

### 2.10. Immunocytochemistry

To test whether tranylcypromine affects LPS-evoked p-STAT3, p-NF-κB, or p-ERK levels, BV2 microglial cells were pretreated with LPS (1 μg/mL) or PBS for 30 min and treated with tranylcypromine (5 μM) or vehicle (1% DMSO) for 5.5 h. Immunocytochemistry was then conducted with anti-CD11b and anti-p-NF-κB antibodies, anti-CD11b and anti-p-STAT3 (Ser727), or anti-CD11b and anti-p-ERK antibodies, as previously described [25,26,27].

### 2.11. Subcellular Fractionation (Cytosol vs. Nuclear)

BV2 microglial cells were used to assess the effects of tranylcypromine on LPS-induced cytosolic and nuclear p-STAT3 levels. After pretreatment with LPS (1 μg/mL) or PBS for 30 min, the cells were treated for 5.5 h with tranylcypromine (5 μM) or vehicle (1% DMSO). Lysis of the treated cells was performed in cytosolic fractionation buffer for 5 min (10 mM HEPES pH 8.0, 1.5 mM MgCl_2_, 10 mM KCl, 0.5 mM DTT, 300 mM sucrose, 0.1% NP-40, and 0.5 mM PMSF), and the cytosolic fraction was obtained as the supernatant after centrifugation at 10,000 rpm and 4 °C for 1 min. The nuclear fraction was obtained by centrifugation at 10,000 rpm at 4 °C for 15 min after lysis of the pellet in nuclear fractionation buffer (10 mM HEPES pH 8.0, 20% glycerol, 100 mM KCl, 100 mM NaCl, 0.2 mM EDTA, 0.5 mM DTT, and 0.5 mM PMSF) on ice for 15 min. The cytosolic and nuclear fractions were analyzed by Western blotting with anti-p-STAT3 (Ser727), anti-β-actin, or anti-PCNA antibodies. Fusion Capt Advance software (Vilber Lourmat) was used for data analysis.

### 2.12. Statistical Analyses

Data analyses were performed using GraphPad Prism 7 software. Unpaired two-tailed T-tests with Welch’s correction were used for comparisons between two groups, and one-way ANOVA was used for multiple comparisons. Post hoc analyses were performed using Tukey’s multiple comparison test with significance set at *p* <0.05. Data for groups in which LPS treatment did not promote proinflammatory cytokine levels or TLR4/MAPK signaling pathways compared to the control were excluded. Data are presented as the mean ± S.E.M. (* *p* < 0.05, ** *p* < 0.01, *** *p* < 0.001).

## 3. Results

### 3.1. Tranylcypromine Regulates LPS-Mediated Proinflammatory Cytokine Levels in BV2 Microglial Cells

**Figure 1:**
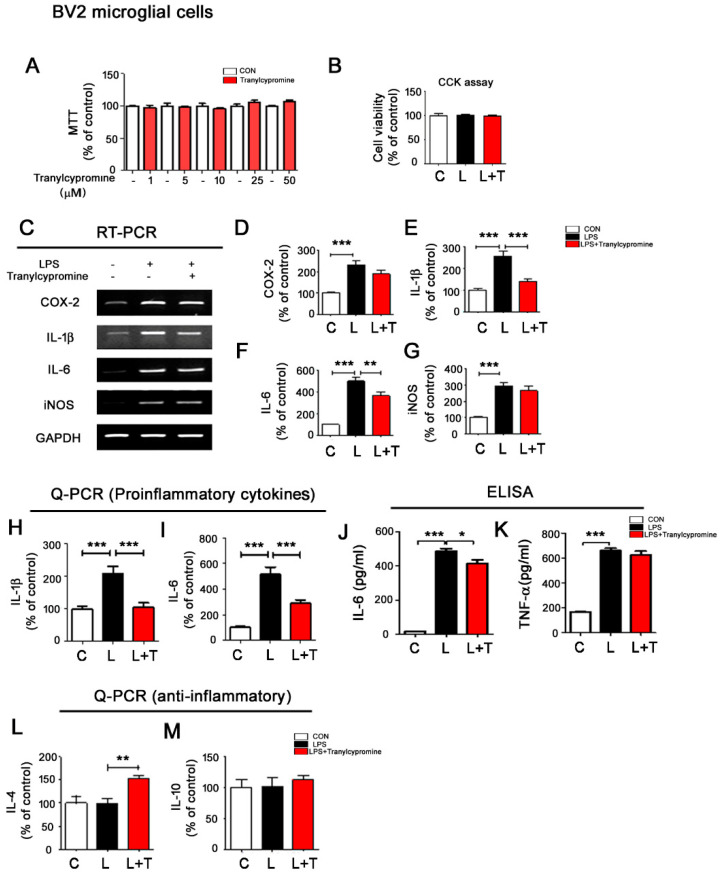
Post treatment with tranylcypromine selectively regulates LPS-evoked proinflammatory cytokine levels in BV2 microglial cells. (**A**) BV2 cells were treated with vehicle (1% DMSO) or tranylcypromine (1, 5, 10, 25, and 50 μM) for 24 h, and cell viability was measured (**A**, *n* = 12 for each dose). (**B**) BV2 microglial cells were pretreated with LPS (1 μg/mL) or PBS for 30 min and treated with vehicle (1% DMSO) or tranylcypromine (5 μM) for 5.5 h, and CCK assays were performed (*n* = 24/group). (**C**–**I**) BV2 microglial cells were pretreated with LPS (1 μg/mL) or PBS for 30 min and treated with vehicle (1% DMSO) or tranylcypromine (5 μM) for 5.5 h, and proinflammatory cytokine levels were measured by RT-PCR (*n* = 16/group) or Q-PCR (IL-1β and IL-6; *n* = 10/group). (**J**,**K**) BV2 microglial cells were pretreated with LPS (200 ng/mL) or PBS for 30 min and treated with vehicle (1% DMSO) or tranylcypromine (5 μM) for 23.5 h, and ELISA was performed (*n* = 8/group). (**L**,**M**) BV2 microglial cells were pretreated with LPS (1 μg/mL) or PBS for 30 min and treated with vehicle (1% DMSO) or tranylcypromine (5 μM) for 5.5 h, and anti-inflammatory cytokine levels were measured by real time-PCR (*n* = 4/group). * *p* < 0.05, ** *p* < 0.01, *** *p* < 0.001. CON and C, control; L, LPS; L + T, LPS + Tranylcypromine.

MTT assays of BV2 microglial cells treated with vehicle (1% DMSO) or tranylcypromine (1, 5, 10, 25, or 50 μM) for 24 h showed that tranylcypromine did not exhibit BV2 cell cytotoxicity up to 50 μM (Figure 1A).

The effects of tranylcypromine on LPS-induced proinflammatory cytokine responses were assessed by pretreating BV2 microglial cells with LPS (200 ng/mL) or PBS for 30 min before treatment with tranylcypromine (1 or 5 μM) or vehicle (1% DMSO) for 5.5 h. Post treatment with 1 μM tranylcypromine did not alter any proinflammatory cytokine levels (Supplementary Materials, Figure S1A–E). However, when the post treatment concentration of tranylcypromine was increased to 5 μM, significant decreases in LPS-mediated proinflammatory cytokine IL-1β and IL-6 mRNA levels were observed (Figure S1A–E). In parallel experiments, BV2 microglial cells were pretreated with tranylcypromine (1 or 5 μM) or vehicle (1% DMSO) for 30 min before treatment with LPS (200 ng/mL) or PBS for 5.5 h. Pretreatment with 5 μM tranylcypromine reduced the level of IL-1β, but not the levels of other proinflammatory cytokines induced by 200 ng/mL LPS (Figure S1F–J).

Next, we tested the ability of the pretreatment with 5 μM tranylcypromine to alter proinflammatory cytokine levels induced by a higher concentration of LPS (1 μg/mL). Before performing these experiments, we first confirmed, using CCK assays, that 1 μg/mL LPS did not exhibit cytotoxicity under the conditions of the tranylcypromine pretreatment experiments (Figure S2A,B). Pretreatment with 5 μM tranylcypromine significantly decreased the level of IL-1β mRNA induced by 1 μg/mL LPS but did not significantly alter other proinflammatory cytokine levels (Figure S2A,C–F). In addition, real-time PCR analysis showed that pretreatment with 5 μM tranylcypromine did not alter anti-inflammatory cytokine levels in these cells (Figure S2G,H).

We then investigated the effect of post treatment with tranylcypromine on the proinflammatory responses induced by a higher concentration of LPS (1 μg/mL). Again, we first confirmed by CCK assay that 1 μg/mL LPS did not exhibit cytotoxicity under the conditions of the tranylcypromine post treatment experiments (Figure 1B). RT-PCR revealed that post treatment with 5 μM tranylcypromine significantly decreased the mRNA levels of IL-1β and IL-6 induced by 1 μg/mL LPS (Figure 1C–G). To further confirm our findings, we conducted real-time PCR and ELISA and found that post treatment with 5 μM tranylcypromine significantly downregulated 1 μg/mL LPS-evoked proinflammatory cytokine IL-1β and IL-6 mRNA levels compared with LPS treatment alone using Q-PCR (Figure 1H,I). Moreover, post treatment with 5 μM tranylcypromine significantly reduced LPS-mediated IL-6 but not TNF-α levels using ELISA (Figure 1J,K). 

To examine the effects of post treatment with tranylcypromine on anti-inflammatory cytokine levels, BV2 microglial cells were pretreated with 1 μg/mL LPS or PBS for 30 min and treated with 5 μM tranylcypromine or vehicle (1% DMSO) for 5.5 h, and anti-inflammatory cytokine levels were measured using Q-PCR. Interestingly, we found that pretreatment with LPS and post treatment with 5 μM tranylcypromine significantly increased anti-inflammatory cytokine IL-4 mRNA levels (Figure 1L,M).

The effects of tranylcypromine on LPS-mediated astrocytic proinflammatory responses were assessed in primary astrocytes. Post treatment with 5 μM tranylcypromine did not alter LPS-induced proinflammatory cytokine and anti-inflammatory cytokine levels in primary astrocytes pretreated with LPS (Figure S3A–G). These data suggest that tranylcypromine modulates LPS-induced microglial proinflammatory responses in BV2 microglial cells but not primary astrocytes.

### 3.2. Tranylcypromine Affects LPS-Induced TLR4/ERK Signaling in BV2 Microglial Cells

**Figure 2:**
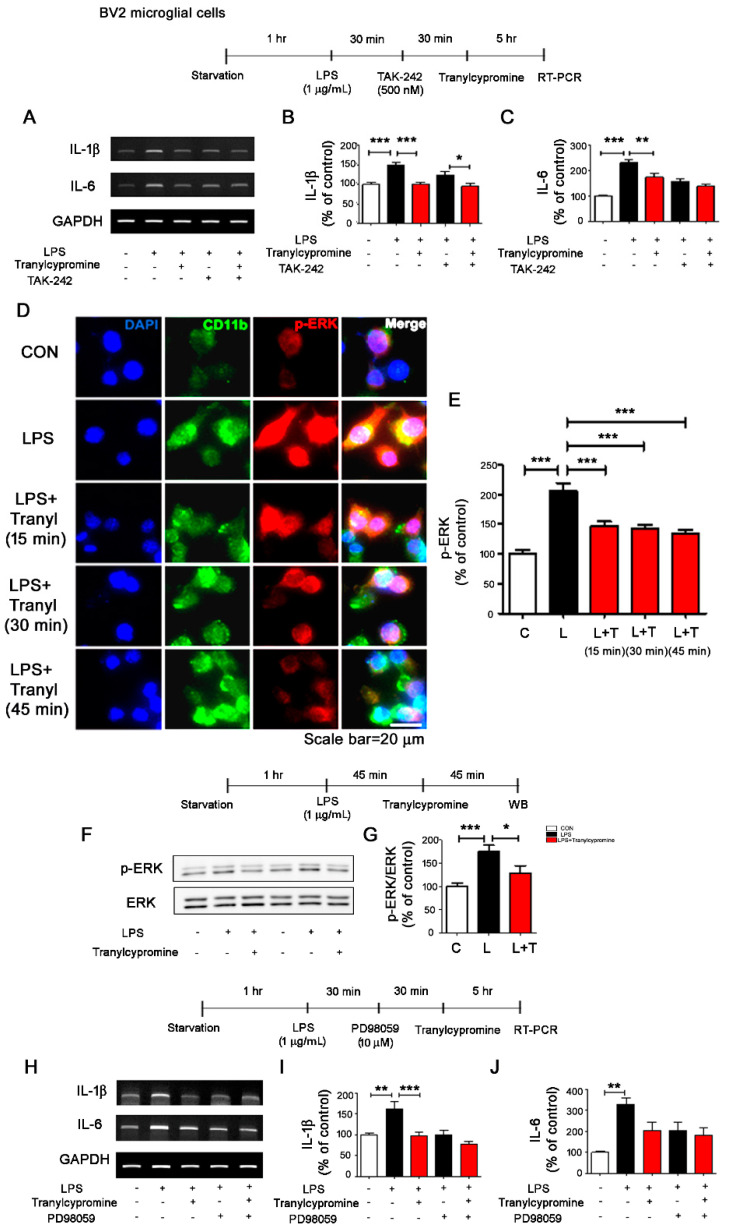
Tranylcypromine inhibits TLR4/ERK signaling in BV2 microglial cells. (**A**–**C**) BV2 microglial cells were pretreated with LPS (1 μg/mL) or PBS for 30 min, treated with TAK-242 (TLR4 inhibitor, 500 nM) for 30 min, and treated with tranylcypromine (5 μM) or PBS for 5 h. IL-1β or IL-6 mRNA levels were then measured using RT-PCR (*n* = 11/group). (**D**–**E**) BV2 microglial cells were pretreated with LPS (1 μg/mL) or PBS for 45 min and treated with tranylcypromine (5 μM) or vehicle (1% DMSO) for 15, 30, or 45 min, and immunocytochemistry was conducted (CON, *n* = 211; LPS, *n* = 241; LPS + tranylcypromine (15 min), *n* = 333; LPS + tranylcypromine (30 min), *n* = 230; LPS + tranylcypromine (45 min), *n* = 314). (**F**,**G**) BV2 microglial cells were pretreated with LPS (1 μg/mL) or PBS for 45 min and treated with tranylcypromine (5 μM) or vehicle (1% DMSO) for 45 min, and Western blotting was performed with anti-p-ERK and anti-ERK antibodies (*n* = 14/group). (**H**–**J**) BV2 microglial cells were pretreated with LPS (1 μg/mL) or PBS for 30 min, treated with ERK inhibitor (PD98059, 10 μM) or vehicle (1% DMSO) for 30 min, and treated with tranylcypromine (5 μM) or vehicle (1% DMSO) for 5 h. IL-1β or IL-6 mRNA levels were then measured using RT-PCR (*n* = 10/group). * *p* < 0.05, ** *p* < 0.01, *** *p* < 0.001. CON and C, control; L, LPS; L + T, LPS + Tranylcypromine.

To determine the molecular mechanism by which tranylcypromine modulates LPS-induced proinflammatory responses, we first investigated whether tranylcypromine inhibits TLR4 signaling to alter LPS-evoked proinflammatory cytokine levels. For these experiments, BV2 microglial cells were pretreated with LPS (1 μg/mL) or PBS for 30 min, treated with TAK-242 (TLR4 inhibitor, 500 nM) or vehicle (1% DMSO) for 30 min, and subsequently, treated with tranylcypromine (5 μM) or vehicle (1% DMSO) for 5 h. Tranylcypromine significantly decreased LPS-induced IL-1β and IL-6 mRNA levels compared to LPS treatment (Figure 2A–C). In addition, treatment with TAK-242, LPS, and tranylcypromine significantly reduced LPS-induced IL-1β mRNA levels compared with treatment with LPS and TAK-242, but not treatment with LPS and tranylcypromine (Figure 2A,B). Moreover, treatment with TAK-242, LPS, and tranylcypromine did not affect LPS-mediated IL-6 mRNA levels compared with treatment with LPS and TAK-242 and treatment with LPS and tranylcypromine (Figure 2A,C). These data indicate that tranylcypromine is dependent on TLR4 signaling to alter LPS-induced IL-6 mRNA levels but only partially dependent on TLR4 signaling to affect LPS-induced IL-1β mRNA levels.

We then examined whether tranylcypromine modulates ERK signaling, which plays an important role in LPS-mediated neuroinflammation in vitro and in vivo [26,28]. For these experiments, BV2 microglial cells pretreated with LPS (1 μg/mL) or PBS for 45 min and treated with tranylcypromine (5 μM) or vehicle (1% DMSO) for 15, 30, or 45 min were subjected to immunocytochemistry with an anti-p-ERK antibody. Tranylcypromine significantly decreased LPS-evoked p-ERK levels compared to LPS treatment (Figure 2D,E). Western blotting with anti-p-ERK and anti-ERK antibodies confirmed that tranylcypromine significantly reduced LPS-mediated p-ERK levels in BV2 microglial cells but did not alter total ERK levels (Figure 2F,G).

To investigate the ERK dependence of the effects of tranylcypromine on LPS-induced proinflammatory cytokine levels, BV2 microglial cells were pretreated with LPS (1 μg/mL) or PBS for 30 min, treated with PD98059 (ERK inhibitor, 10 μM) or vehicle (1% DMSO) for 30 min, and subsequently, treated with tranylcypromine (5 μM) or vehicle (1% DMSO) for 5 h. Treatment with PD98059, LPS, and tranylcypromine did not significantly alter LPS-induced IL-1β or IL-6 mRNA levels compared with treatment with LPS and PD98059 or LPS and tranylcypromine (Figure 2H–J), indicating that tranylcypromine modulates ERK signaling to modify LPS-induced microglial proinflammatory cytokine levels in BV2 microglial cells.

### 3.3. Tranylcypromine Regulates LPS-Evoked Microglial Nuclear p-STAT3 and p-NF-κB Levels

**Figure 3:**
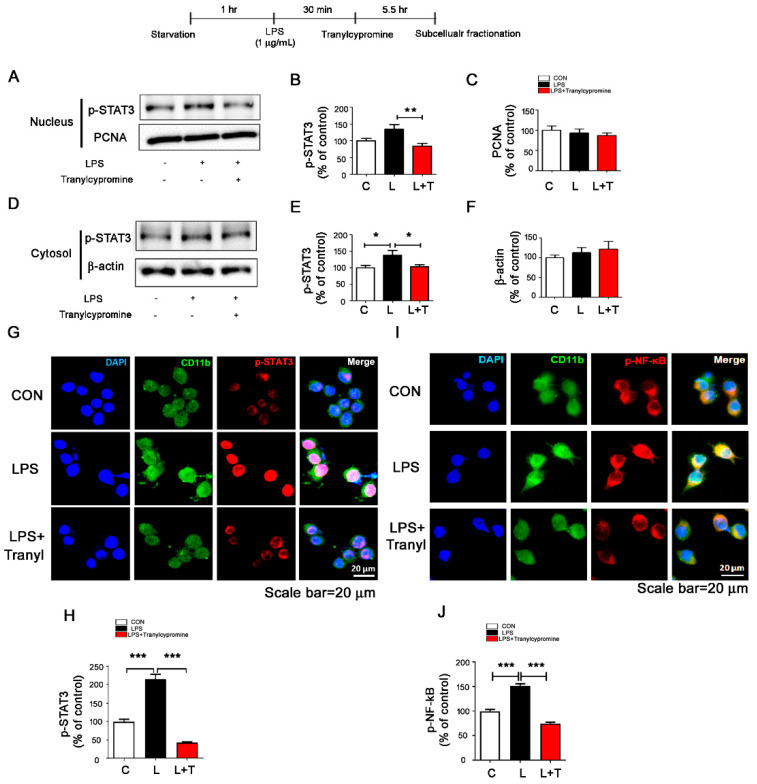
Tranylcypromine treatment downregulates LPS-mediated nuclear p-STAT3 levels in BV2 microglial cells. (**A**–**F**) BV2 microglial cells were pretreated with LPS (1 μg/mL) or PBS for 30 min and treated with tranylcypromine (5 μM) or vehicle (1% DMSO) for 5.5 h before subcellular fractionation (Nucleus and cytosol: *n* = 27/group). (**G**–**J**) BV2 microglial cells were pretreated with LPS (1 μg/mL) or PBS for 30 min and treated with tranylcypromine (5 μM) or vehicle (1% DMSO) for 5.5 h. Immunocytochemistry was then performed with anti-CD11b and anti-p-STAT3 (s727) or anti-CD11b and anti-p-NF-κB antibodies (p-STAT3: CON, *n* = 257; LPS, *n* = 260; LPS + tranylcypromine, *n* = 293; p-NF-κB: CON, *n* = 240; LPS, *n* = 308; LPS + tranylcypromine, *n* = 325). * *p* < 0.05, ** *p* < 0.01, *** *p* < 0.001. CON and C, control; L, LPS; L + T, LPS + Tranylcypromine.

Next, we attempted to identify the transcription factor involved in the regulation of LPS-induced neuroinflammatory responses by tranylcypromine. Subcellular fractionation of BV2 microglial cells treated successively with 1 μg/mL LPS and 5 μM tranylcypromine revealed that tranylcypromine significantly decreased LPS-induced microglial cytosol and nuclear p-STAT3 levels compared to LPS treatment (Figure 3A–F). To further confirm our findings, we conducted immunocytochemistry with anti-CD11b and anti-p-STAT3 (Ser727) antibodies and found that tranylcypromine significantly reduced LPS-mediated nuclear p-STAT3 levels (Figure 3G,H). We then examined whether tranylcypromine alters LPS-induced levels of NF-κB, a traditional transcription factor in neuroinflammatory responses. Interestingly, we found that tranylcypromine also significantly suppressed LPS-mediated nuclear p-NF-κB levels in BV2 microglial cells (Figure 3I,J). These data suggest that tranylcypromine alters LPS-evoked microglial nuclear p-STAT3 and p-NF-κB levels to regulate neuroinflammatory responses in vitro.

### 3.4. Tranylcypromine Treatment Decreases LPS-Mediated Microglial Activation in Wild-Type Mice

**Figure 4:**
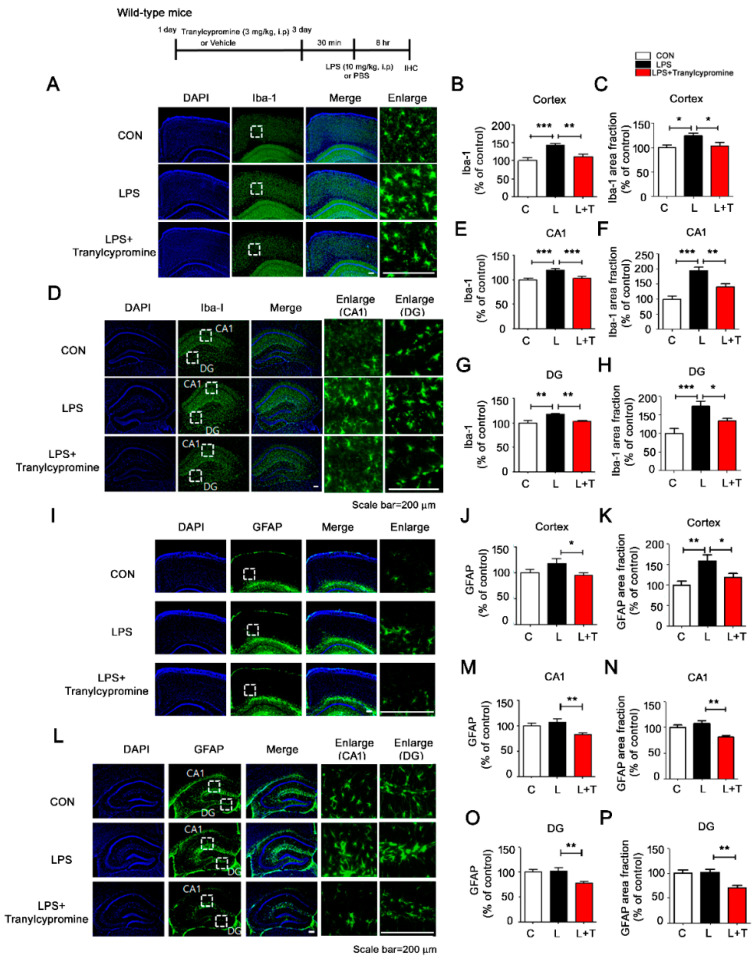
Tranylcypromine suppresses LPS-mediated microgliosis in wild-type mice. (**A**,**D**,**I**,**L**) After tranylcypromine (3 mg/kg, i.p.) or PBS injection daily for 3 days, wild-type mice were injected with LPS (10 mg/kg, i.p.) or PBS. Perfused and fixed mice were then subjected to immunohistochemistry with anti-Iba-1 or anti-GFAP antibodies. (**B**,**C**) Quantification of Iba-1 intensity and area fraction from A (cortex: con, *n* = 4 mice; LPS, *n* = 5 mice; tranylcypromine, *n* = 5 mice). (**E**–**H**) Quantification of Iba-1 intensity and area fraction from D (CA1 and DG; con, *n* = 4 mice; LPS, *n* = 5 mice; tranylcypromine, *n* = 5 mice). (**J**,**K**) Quantification of GFAP intensity and area fraction from I (cortex: *n* = 5 mice/group). (**M**–**P**) Quantification of GFAP intensity and area fraction from L (CA1 and DG; *n* = 5 mice/group). * *p* < 0.05, ** *p* < 0.01, *** *p* < 0.001. CON and C, control; L, LPS; L + T, LPS + Tranylcypromine.

Since tranylcypromine altered LPS-induced neuroinflammatory responses in vitro, we examined whether tranylcypromine regulates LPS-induced microglial and astrocyte activation in vivo. Based on the literature, we selected a tranylcypromine dose of 3 mg/kg for this study [29,30,31]. Wild-type mice were injected with tranylcypromine (3 mg/kg, i.p.) or PBS daily for 3 days and subsequently injected with LPS (10 mg/kg, i.p.) or PBS. After 8 h, the mice were perfused and fixed, and immunohistochemistry was conducted with anti-Iba-1 or anti-GFAP antibodies. Tranylcypromine significantly downregulated LPS-stimulated microglial activation in the cortex and hippocampus (Figure 4A–H). However, tranylcypromine significantly downregulated LPS-induced astrocyte activation only in the cortex (Figure 4I–P), suggesting that the effects of tranylcypromine on LPS-mediated microgliosis are greater than those on astrogliosis in wild-type mice. These data indicate that tranylcypromine modulates LPS-induced microglial activation in wild-type mice. 

### 3.5. Tranylcypromine Treatment Reduces LPS-Mediated Proinflammatory Cytokine COX-2 and IL-6 Levels in Wild-Type Mice

**Figure 5:**
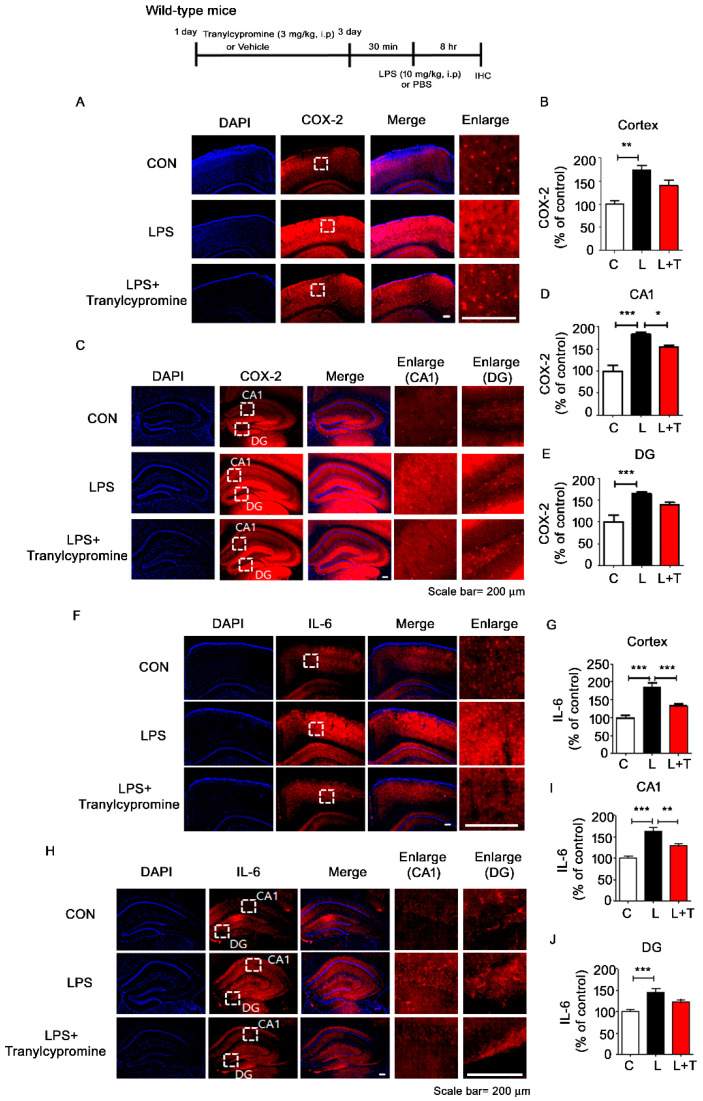
Tranylcypromine decreases LPS-mediated proinflammatory cytokine COX-2 and IL-6 levels in wild-type mice. (**A**,**C**,**F**,**H**) After daily injection with tranylcypromine (3 mg/kg, i.p.) or PBS for 3 days, wild-type mice were injected with LPS (10 mg/kg, i.p.) or PBS. Perfused and fixed mice were then subjected to immunohistochemistry with anti-COX-2 or anti-IL-6 antibodies. (**B**,**D**,**E**) Quantification of data from (**A**) (cortex: con, *n* = 4 mice; LPS, *n* = 5 mice; tranylcypromine, *n* = 5 mice) and **C** (CA1 and DG: con, *n* = 4 mice; LPS, *n* = 5 mice; tranylcypromine, *n* = 5 mice). (**G**,**I**,**J**) Quantification of data from (**F**) (cortex: con, *n* = 4 mice; LPS, *n* = 5 mice; tranylcypromine, *n* = 5 mice) and (**H**) (CA1 and DG: con, *n* = 4 mice; LPS, *n* = 5 mice; tranylcypromine, *n* = 5 mice). * *p* < 0.05, ** *p* < 0.01, *** *p* < 0.001. CON and C, control; L, LPS; L + T, LPS + Tranylcypromine.

**Figure 6:**
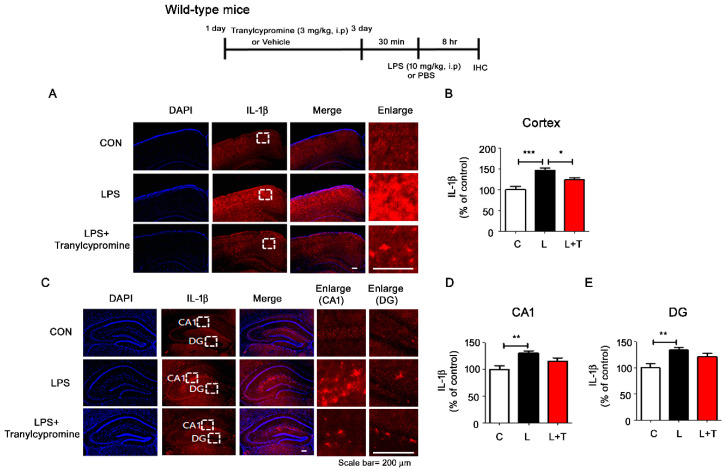
Tranylcypromine downregulates LPS-mediated proinflammatory cytokine IL-1β levels in the cortex in wild-type mice. (**A**–**E**) After daily injection with tranylcypromine (3 mg/kg, i.p.) or PBS for 3 days, wild-type mice were injected with LPS (10 mg/kg, i.p.) or PBS. Perfused and fixed mice were then subjected to immunohistochemistry with an anti-IL-1β antibody (cortex, hippocampus CA1 and DG: con, *n* = 4 mice; LPS, *n* = 5 mice; tranylcypromine, *n* = 5 mice). * *p* < 0.05, ** *p* < 0.01, *** *p* < 0.001. CON and C, control; L, LPS; L + T, LPS + Tranylcypromine.

To investigate whether tranylcypromine affects LPS-mediated proinflammatory cytokine levels in vivo, wild-type mice were injected with tranylcypromine (3 mg/kg, i.p.) or PBS daily for 3 days and subsequently injected with LPS (10 mg/kg, i.p.) or PBS. COX-2, IL-1β, and IL-6 levels were upregulated in LPS-injected wild-type mice compared with vehicle-injected wild-type mice (Figure 5 and Figure 6). These increases were reduced in LPS-injected wild-type mice pretreated with tranylcypromine, although the magnitude of these reductions varied depending on the region of the brain. Tranylcypromine injection reduced LPS-induced COX-2 levels in hippocampus CA1 but not the cortex and hippocampus DG (Figure 5A–E); decreased LPS-evoked IL-6 levels in the cortex and hippocampus CA1 but not hippocampus DG (Figure 5F–J); and suppressed LPS-mediated IL-1β levels in the cortex but not in hippocampus CA1 and DG (Figure 6A–E). 

We then examined whether the proinflammatory cytokine IL-1β colocalized with microglia and/or neuronal cells in vivo. For these experiments, wild-type mice were injected with tranylcypromine (3 mg/kg, i.p.) or PBS daily for 3 days and subsequently injected with LPS (10 mg/kg, i.p.) or PBS. Immunohistochemistry with anti-IL-1β and anti-CD11b or anti-IL-1β and anti-NeuN antibodies showed that the proinflammatory cytokine IL-1β colocalized mostly with microglial cells in LPS-treated wild-type mice and not neuronal cells (Figures S4 and S5). These data suggest that tranylcypromine differentially alters LPS-stimulated proinflammatory cytokine levels in wild-type mice.

### 3.6. Tranylcypromine-Injected 5xFAD Mice Exhibit Downregulated Aβ-Mediated Microglial Activation

**Figure 7:**
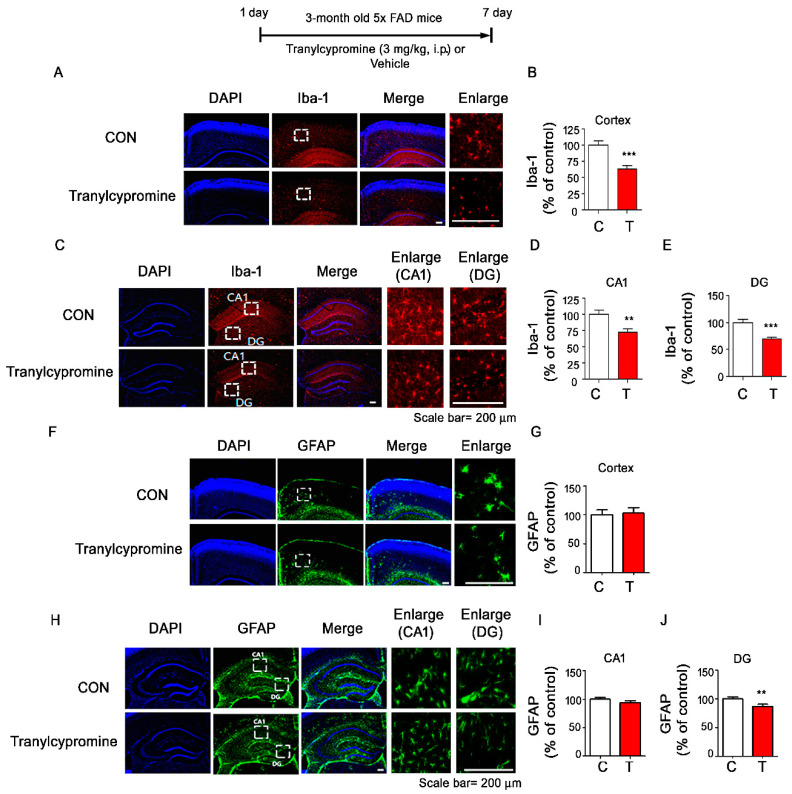
Tranylcypromine alters Aβ-induced microgliosis in a mouse model of AD. (**A**,**C**,**F**,**H**) 5xFAD mice were injected with tranylcypromine (3 mg/kg, i.p.) or PBS daily for 7 days, and immunohistochemistry was performed with anti-Iba-1 and anti-GFAP antibodies. (**B**,**D**,**E**) Quantification of data from (**A**) (cortex: *n* = 5 mice/group) and (**C**) (CA1 and DG: *n* = 5 mice/group). (**G**,**I**,**J**) Quantification of data from (**F**) (cortex: *n* = 5 mice/group) and (**H**) (CA1 and DG: *n* = 5 mice/group) ** *p* < 0.01, *** *p* < 0.001. CON and C, control; T, Tranylcypromine.

To investigate whether tranylcypromine modulates Aβ-mediated gliosis in a mouse model of AD, 3-month-old 5xFAD mice injected with tranylcypromine (3 mg/kg, i.p.) or PBS daily for 7 days were subjected to immunohistochemistry with anti-Iba-1 and anti-GFAP antibodies. Tranylcypromine injection significantly inhibited Aβ-mediated microglial activation in the cortex or hippocampus in 5xFAD mice (Figure 7A–E). However, tranylcypromine significantly downregulated Aβ-induced astrocyte activation only in the hippocampus DG and not in the cortex and hippocampus CA1 (Figure 7F–J), suggesting that tranylcypromine differentially regulates microglial and astrocyte activation in this mouse model of AD.

## 4. Discussion

In the present study, we examined the effects of the MAO inhibitor tranylcypromine on neuroinflammatory responses in vitro and in vivo. We found that tranylcypromine selectively suppressed LPS-induced IL-1β and IL-6 mRNA levels in BV2 microglial cells by inhibiting LPS-mediated TLR4/ERK/STAT3 signaling. Strikingly, in wild-type mice, tranylcypromine injection reduced both LPS-induced gliosis and proinflammatory cytokine levels. Moreover, in 5xFAD mice, tranylcypromine significantly downregulated Aβ-mediated microglial activation in 5xFAD mice but had smaller effects on astrocyte activation. Taken together, our results indicate that tranylcypromine regulates LPS- and Aβ-mediated neuroinflammatory responses in BV2 microglial cells, wild-type mice, and a mouse model of AD.

### 4.1. Tranylcypromine Alters LPS-Induced Proinflammatory Cytokine Production

Several recent studies have demonstrated that MAO plays an important role in cardiovascular oxidative stress (e.g., ROS) and induction of inflammation by promoting endothelial dysfunction [32,33]. In addition, MAO-A expression, activity, and function are altered in IL-13-induced monocytes and in A549 lung carcinoma cells [34]. Vega et al. found that MAO alters macrophage-inducible nitric oxide synthase gene expression [35]. Thus, modulating MAO expression and its activity is a potential therapeutic strategy for cancer, neuroinflammation-related diseases, and endothelial dysfunction-related diseases. Indeed, Ratiu et al. found that reversible and irreversible MAO inhibitors improve vascular function and reduce oxidative stress in LPS-induced acute inflammation [36]. Additionally, moclobemide, a reversible selective MAO inhibitor, reduces proinflammatory cytokine (IL-1β and TNF-α) levels in LPS-stimulated primary rat mixed glial cell cultures [36]. However, another MAO inhibitor, phenelzine, increases LPS-induced inducible nitric oxide synthase (iNOS), TNF-α, and IL-6 levels in primary microglial cells, suggesting that the direction of the effects of MAO inhibition on inflammatory responses depends on the dosage and type of MAO inhibitor (selective or non-selective/reversible or irreversible) [37,38,39]. In this study, we tested whether tranylcypromine, which has less adverse effects than other MAO inhibitors in patients with depression, can alter LPS-stimulated neuroinflammatory responses. We found that 5 μM tranylcypromine was more effective than 1 μM in lowering proinflammatory cytokine levels induced by LPS (200 ng/mL and 1 μg/mL) in BV2 microglial cells (Figure 1, Figures S1 and S2). In addition, post treatment with tranylcypromine (as a curative condition) was more effective in reducing LPS-evoked microglial proinflammatory responses than pretreatment (as a preventive condition) (Figure 1, Figure S2). On the other hand, tranylcypromine had no effect on LPS-induced proinflammatory levels in primary astrocytes (Figure S3). Our findings suggest that tranylcypromine differentially modulates LPS-induced microglial proinflammatory and anti-inflammatory cytokine production depending on the cell type and experimental procedure (i.e., concentration of tranylcypromine, pre vs. post treatment, LPS concentration).

### 4.2. Tranylcypromine Regulates LPS-Induced Microglial Neuroinflammatory Signaling Pathways

TLRs, a key component of innate immune signaling, are the predominant receptors involved in the LPS-mediated immune response. In response to LPS, TLR4 with its coreceptor, cluster of differentiation 14 (CD 14), releases various proinflammatory cytokines during the inflammation process [40]. There have been no reports on whether MAO or MAO inhibitors regulate TLR4 signaling to alter LPS-induced microglial proinflammatory cytokine levels. In this study, we found that tranylcypromine suppressed TLR4 signaling to downregulate LPS-induced IL-6 mRNA levels in a TLR4-dependent manner. However, tranylcypromine downregulated LPS-induced IL-1β mRNA levels in a partially TLR4-independent manner (Figure 2). These data suggest that tranylcypromine alters other neuroinflammation-related signaling pathways (e.g., MAPK and/or AKT signaling) to regulate LPS-induced microglial proinflammatory cytokine IL-1β or IL-6 levels. Interestingly, a recent study found that MAO-B induces MAPK and ERK activity as well as cell mitogenesis in HEK293 cells [41]. Here, we found that tranylcypromine, which irreversibly inhibits MAO-A and MAO-B, significantly inhibited ERK activation to decrease LPS-induced IL-6 or IL-1β mRNA levels in BV2 microglial cells (Figure 2). These results suggest that MAO and/or MAO inhibitors affect ERK signaling to alter LPS-induced microglial proinflammatory responses. Future studies will examine whether other MAO inhibitors can regulate TLR4/MAPK signaling to modulate LPS-mediated neuroinflammatory responses and compare their effects with those of tranylcypromine.

The transcription factor STAT3 mediates cell growth and differentiation and plays a central role in transmitting signals from the membrane to the nucleus through the IL-6 family of cytokine receptors [42]. STAT3 is activated by IL-6 in transient inflammatory and proinflammatory states but is activated by IL-10 in long-term inflammatory and anti-inflammatory states; consequently, STAT3 acts as an intersection of pro and anti-inflammatory effects [43]. Recently, Dhabal et al. reported that MAO-A gene expression and activity are regulated by STAT1, STAT3, and STAT6 [34]. Thus, we anticipated that MAO inhibitor treatment might modulate the STAT signaling pathway to alter LPS-induced microglial and astrocytic inflammatory responses. Interestingly, we found that tranylcypromine significantly suppressed LPS-induced cytosolic and nuclear STAT3 activation in BV2 microglial cells (Figure 3). Tranylcypromine treatment also downregulated LPS-induced nuclear NF-κB activation in BV2 microglial cells (Figure 3). Future studies will address how tranylcypromine alters both STAT3 and NF-κB signaling to alter LPS-evoked proinflammatory cytokine levels. Overall, our results suggest that tranylcypromine affects TLR4 signaling through the IL-6 family of cytokine receptors as well as downstream ERK and STAT3/NF-κB signaling, to affect LPS-induced microglial proinflammatory responses.

### 4.3. Tranylcypromine Inhibits LPS-Induced Microglial Neuroinflammatory Responses in Wild-Type Mice

Single and/or chronic injections of LPS significantly promote microglial and astrocyte activation and increase proinflammatory cytokine levels in wild-type mice [44]. For instance, a single injection of LPS induces robust expression of the proinflammatory cytokines IL-1β and COX-2 in the hippocampus of wild-type mice [45]. Ratju et al. reported increased endothelial dysfunction and induction of inflammation in LPS-injected rats; treatment of these rats with reversible and irreversible MAO inhibitors improved vascular function and reduced oxidative stress [36]. However, whether the MAO inhibitor tranylcypromine regulates LPS-induced glial activation and proinflammatory cytokine levels has not been established. In this study, we found that tranylcypromine inhibited LPS-induced microglial activation in wild-type mice but had smaller effects on astrocyte activation (Figure 4). In addition, tranylcypromine reduced proinflammatory cytokine COX-2 and IL-1β levels in LPS-injected wild-type mice (Figure 5 and Figure 6). The dose of 3 mg/kg tranylcypromine and daily injection for 3 days and/or LPS injection time frame might not have been sufficient to observe effects of tranylcypromine on LPS-induced astrocyte activation in wild-type mice. In future studies, we will increase the injection time frame and/or dose of tranylcypromine for LPS-treated wild-type mice. Overall, our results suggest that tranylcypromine is a potential therapeutic agent for neuroinflammation-related disease.

### 4.4. Tranylcypromine Modulates the Aβ-Induced Neuroinflammatory Response in 5xFAD Mice

Numerous studies have demonstrated that Aβ oligomer and amyloid fibrils induce microglial and astrocyte activation and proinflammatory cytokine and chemokine production through the amylin receptor [46,47]. In addition, several studies have demonstrated that MAO inhibitors regulate Aβ-induced cell toxicity, microgliosis, and astrogliosis. For example, Park et al. recently reported that injection of the newly synthesized reversible MAO-B inhibitor KDS2010 in APP/PS1 mice (a mouse model of AD) significantly suppresses astrocytic GABA upregulation and astrogliosis and rescues learning and memory [48]. Interestingly, a recent study reported that tranylcypromine has neuroprotective effects against Aβ_1-42_-induced neuronal cell death and neurodegeneration in primary rat cortical neurons [24]. However, whether tranylcypromine alters Aβ oligomer-induced neuroinflammatory responses in vitro and in vivo is unknown. In this study, we found that injection of the MAO inhibitor tranylcypromine markedly reduced microgliosis in 5xFAD mice but had smaller effects on astrogliosis (Figure 7), suggesting that the alleviatory effect of tranylcypromine is specific to microglial cells. Of course, it is possible that injection of 3 mg/kg tranylcypromine daily for 1 week was insufficient in terms of dose or duration to observe effects of tranylcypromine on astrocyte activation. To address this, future studies will determine the molecular mechanisms involved in the tranylcypromine-induced neuroinflammatory response in a mouse model of AD.

### 4.5. Potential Therapeutic Effects of Tranylcypromine on Neurodegenerative Diseases and Future Directions

Inhibitors of MAO (both MAO-A and MAO-B) are currently used in the treatment of depression and anxiety. The MAO inhibitor isocarboxazid is used as an antidepressant and for the treatment of other dementia-related disorders [49]. In mice, injection of the MAO inhibitor phenelzine increases GABA levels in the brain [50]. Interestingly, MAO-B activity increases in an age-dependent manner, and thus, inhibition of MAO-B activity may be a great target for drugs for age-associated human diseases. Consistent with this notion, a recent study suggested that the MAO inhibitor tranylcypromine may have therapeutic effects for AD or PD [51]. Importantly, minimal risk of adverse effects has been observed in elderly depression patients treated with tranylcypromine, supporting the use of this drug for neurodegenerative diseases and/or neuroinflammation-associated diseases [52]. Based on the literature and our findings, future studies could investigate the effects of acute or chronic tranylcypromine injection on AD- and/or PD-related pathophysiological progression. In addition, novel molecular targets of tranylcypromine in AD or PD pathology in specific cell types (i.e., neurons, astrocytes, or microglia) could be identified by RNA sequencing. 

## 5. Conclusions

Tranylcypromine affects LPS-induced neuroinflammatory responses in BV2 microglial cells but not primary astrocytes and inhibits LPS-induced downstream ERK and STAT3 signaling to modulate neuroinflammatory responses. In addition, tranylcypromine inhibits microgliosis as well as proinflammatory cytokine levels in LPS-injected wild-type mice. Moreover, tranylcypromine significantly reduces microglial activation in 5xFAD mice but has smaller effects on astrocyte activation. Taken together, our results indicate that tranylcypromine differentially regulates LPS- and Aβ-induced neuroinflammatory responses in vitro and in vivo.

## Supplementary Materials

The following are available online at https://www.mdpi.com/2073-4409/9/9/1982/s1: Figure S1. Post treatment with 5 μM tranylcypromine significantly decreases LPS-induced proinflammatory cytokine IL-1β and IL-6 levels in BV2 microglial cells; Figure S2. Pretreatment with 5 μM tranylcypromine only significantly reduces LPS-mediated proinflammatory cytokine IL-1β levels in BV2 microglial cells; Figure S3. Post treatment with 5 μM tranylcypromine does not alter LPS-mediated proinflammatory cytokine levels in primary astrocytes; Figure S4. The proinflammatory cytokine IL-1β colocalizes with the microglial cell marker CD11b in the cortex and hippocampus in LPS-treated wild-type mice; Figure S5. The proinflammatory cytokine IL-1β does not colocalize with the neuronal cell marker NeuN in the cortex and hippocampus in LPS-injected wild-type mice.
