# Supplementary material for: The MAO Inhibitor Tranylcypromine Alters LPS- and Aβ-Mediated Neuroinflammatory Responses in Wild-type Mice and a Mouse Model of AD
Source: Cells. 2020 Aug 28;9(9):1982. doi: 10.3390/cells9091982 (PMC7563969; doi:10.3390/cells9091982)
Supplement: Supplementary file 1 [file cells-09-01982-s001.pdf]

The MAO inhibitor tranylcypromine alters LPS- and Aβ-mediated neuroinflammatory responses in wild-type mice and a mouse model of AD

HyunHee Park[1,†], Kyung-Min Han[1,2,†], Hyongjun Jeon[1,†], Ji-Soo Lee[1,†], Hyunju Lee[1], Seong Gak Jeon[1], Jin-Hee Park[1], Yu Gyung Kim[1,6], Yuxi Lin[3], Young-Ho Lee[3,4,5], Yun Ha Jeong [1,*], Hyang-Sook Hoe[1, 2,*]

[1]Department of Neural Development and Disease, Korea Brain Research Institute (KBRI), 61, Cheomdan-ro, Dong-gu, Daegu, Korea, 41062; [2]Department of Brain and Cognitive Sciences, Daegu Gyeongbuk Institute of Science & Technology, Daegu, Korea 42988; [3]Division of Bioconvergence Analysis, Korea Basic Science Institute (KBSI), Ochang, Cheongju, Chungbuk, Korea, 28199; [4]Neurovascular Research Group, Korea Brain Research Institute (KBRI), 61, Cheomdan-ro, Dong-gu, Daegu, Korea, 41062; [5]Bio-Analytical Science, University of Science and Technology (UST), Gajeong-ro, Yuseong-gu, Daejeon, Korea, 34113; [6]Department of Pharmacology, School of Dentistry, Kyungpook National University, Daegu, Korea, 41940; [+]These authors contributed equally to this work.

[*]Corresponding authors:

Hyang-Sook Hoe, Ph.D., Department of Neural Development and Disease, Korea Brain Research Institute (KBRI), 61, Cheomdan-ro, Dong-gu, Daegu, Korea, 41062; E-mail: sookhoe72@kbri.re.kr

Yun Ha Jeong, Ph.D.: Department of Neural Development and Disease, Korea Brain Research Institute (KBRI), 61, Cheomdan-ro, Dong-gu, Daegu, Korea, 41062; E-mail: yunha.jeong@kbri.re.kr

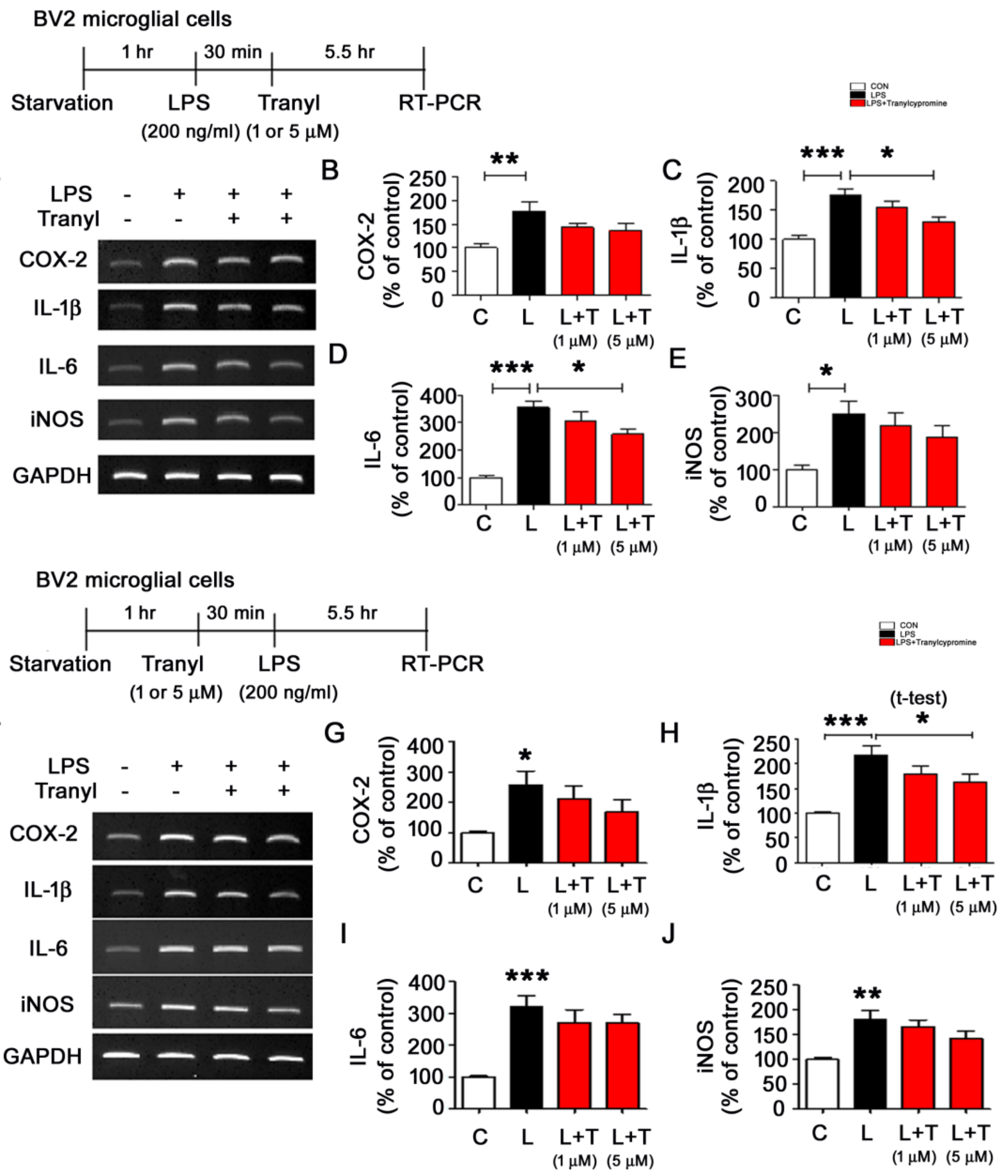

Supplementary Figure S1. Post-treatment with 5 μM tranylcypromine significantly decreases LPS-induced proinflammatory cytokine IL-1β and IL-6 levels in BV2 microglial cells. **(A-E)** Proinflammatory cytokine mRNA levels in BV2 microglial cells pretreated with LPS (200 ng/ml) or PBS for 30 min and treated with vehicle (1% DMSO) or 1 μM or 5 μM tranylcypromine for 5.5 hr (n=5/group) (**F-J**) Proinflammatory cytokine mRNA levels in BV2 microglial cells pretreated with vehicle (1% DMSO) or 1 μM or 5 μM tranylcypromine for 30 min and treated with LPS (200 ng/ml) or PBS for 5.5 h (COX-2, IL-6, and iNOS, n=5/group; IL-1β, n=15/group). *$p < 0.05$, **$p < 0.01$, ***$p < 0.001$

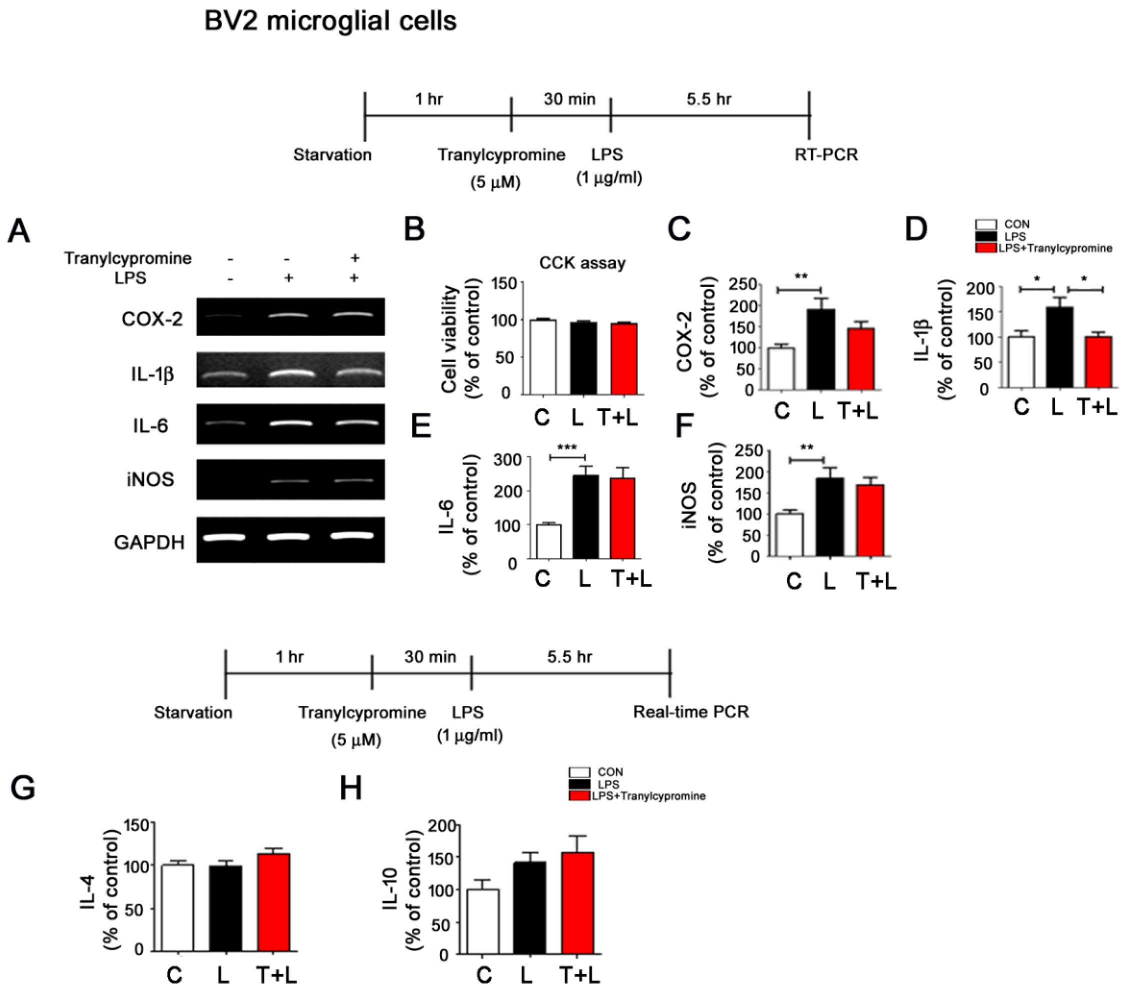

Supplementary Figure S2. Pretreatment with 5 μM tranylcypromine only significantly reduces LPS-mediated proinflammatory cytokine IL-1β levels in BV2 microglial cells. (**A-B**) Cell viability of BV2 microglial cells pretreated with vehicle (1% DMSO) or tranylcypromine (5 μM) for 30 min and treated with LPS (1 μg/mL) or PBS for 5.5 hr (n=24/group). (**C-F**) Proinflammatory cytokine mRNA levels in BV2 microglial cells pretreated with vehicle (1% DMSO) or tranylcypromine (5 μM) for 30 min and treated with LPS (1 μg/mL) or PBS for 5.5 hr (COX-2, IL-1β, and iNOS; n=11/group. IL-6; n=19/group). (**G-H**) Anti-inflammatory cytokine mRNA levels in BV2 microglial cells pretreated with vehicle (1% DMSO) or tranylcypromine (5 μM) for 30 min and treated with LPS (1 μg/mL) or PBS for 5.5 hr (n=8/group).

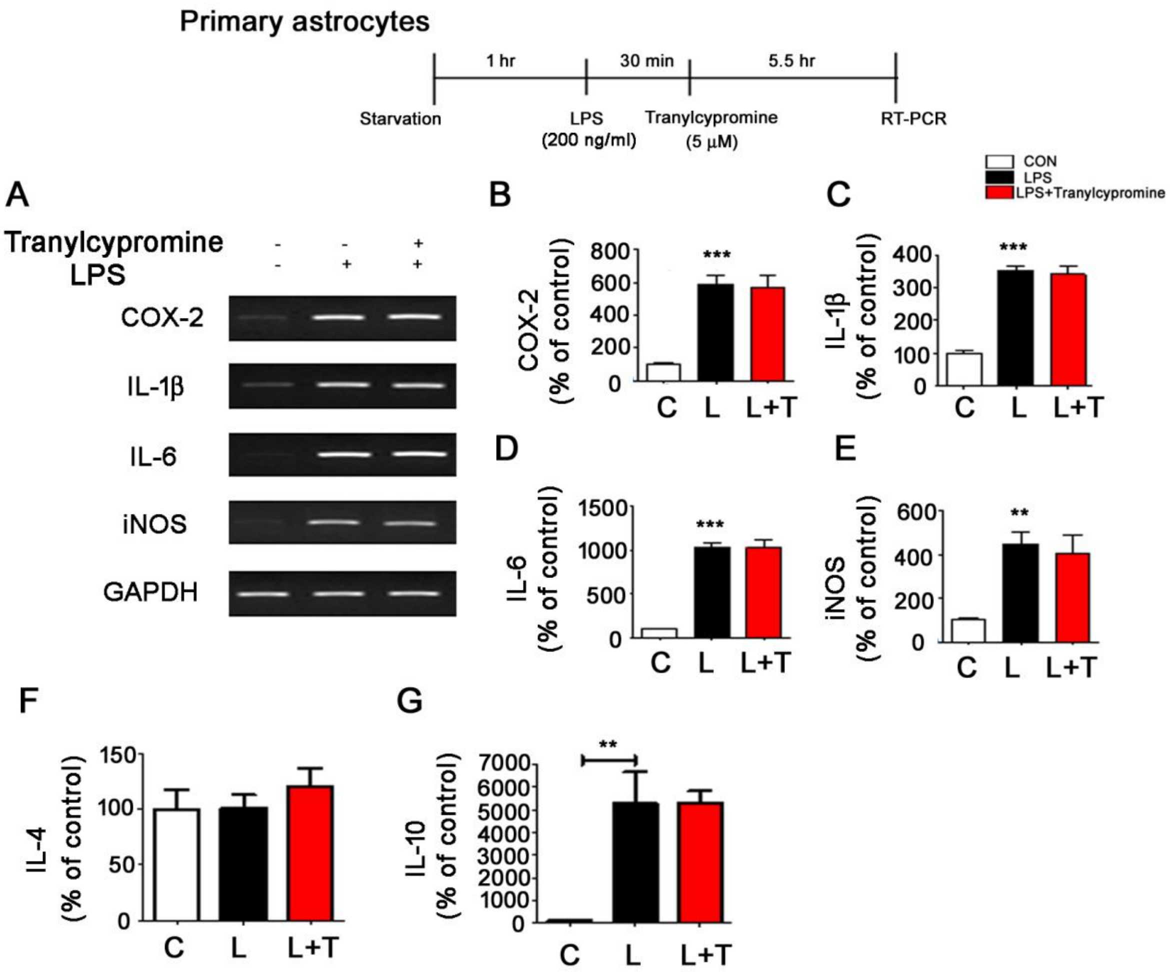

Supplementary Figure S3. Post-treatment with 5 μM tranylcypromine does not alter LPS-mediated proinflammatory cytokine levels in primary astrocytes. (**A**-**E**) Proinflammatory cytokine mRNA levels in primary astrocytes pretreated with LPS (200 ng/ml) or PBS for 30 min and treated with vehicle (1% DMSO) or tranylcypromine (5 μM) for 5.5 h using RT-PCR (COX-2, IL-1β, IL-6, and iNOS; n=4/group). (**F**-**G**) Anti-inflammatory cytokine mRNA levels in primary astrocytes pretreated with LPS (200 ng/ml) or PBS for 30 min and treated with vehicle (1% DMSO) or tranylcypromine (5 μM) for 5.5 hr using real-time PCR (IL-4 and IL-10: n=4/group). **$p < 0.01$, ***$p < 0.001$

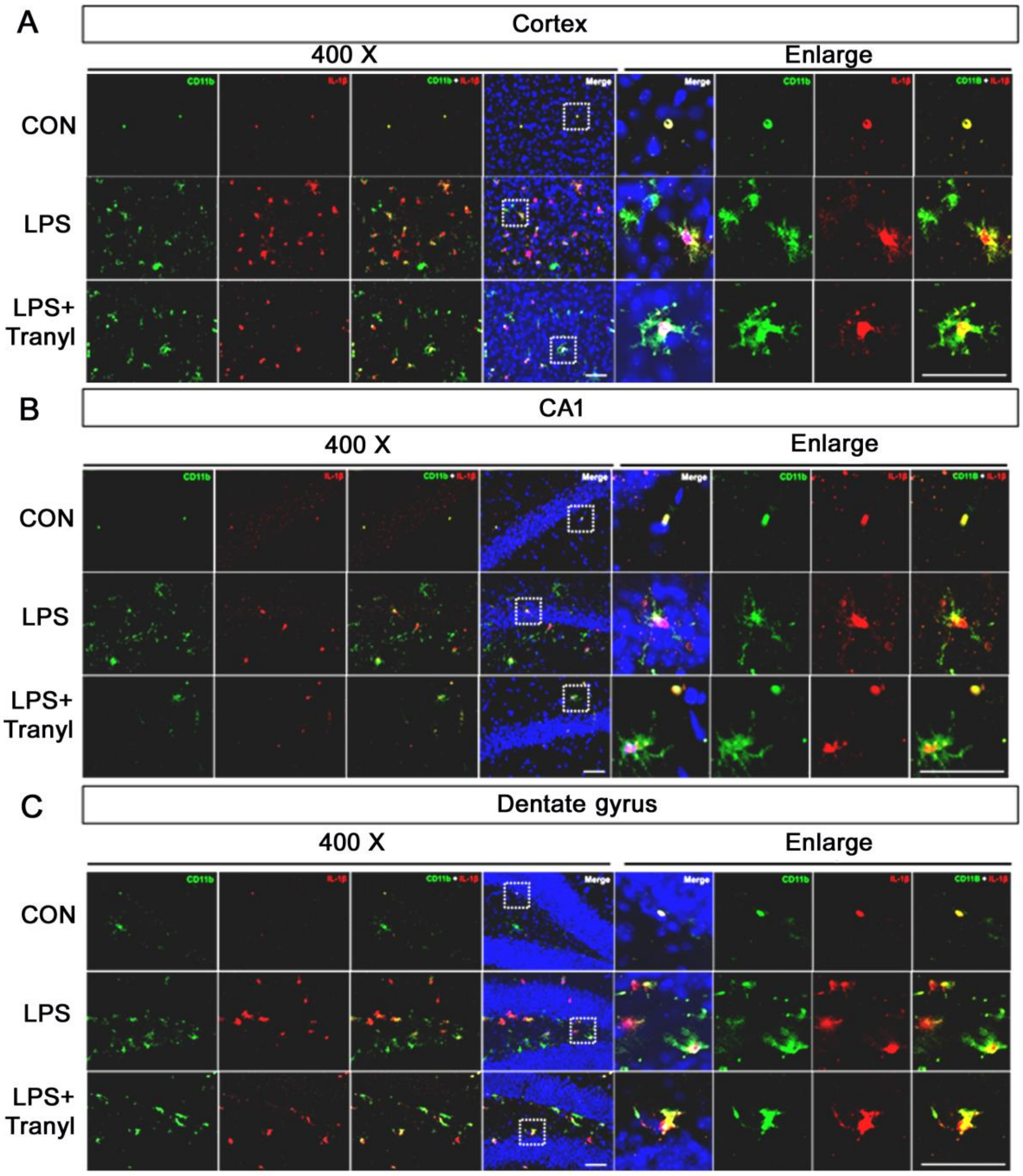

Supplementary Figure S4. The proinflammatory cytokine IL-1β colocalizes with the microglial cell marker CD11b in the cortex and hippocampus in LPS-treated wild-type mice. (**A-C**) After daily injection with tranylcypromine (3 mg/kg, i.p.) or PBS for 3 days, wild-type mice were injected with LPS (10 mg/kg, i.p.) or PBS. Perfused and fixed mice were then subjected to immunohistochemistry with anti-IL-1β and anti-CD11b antibodies.

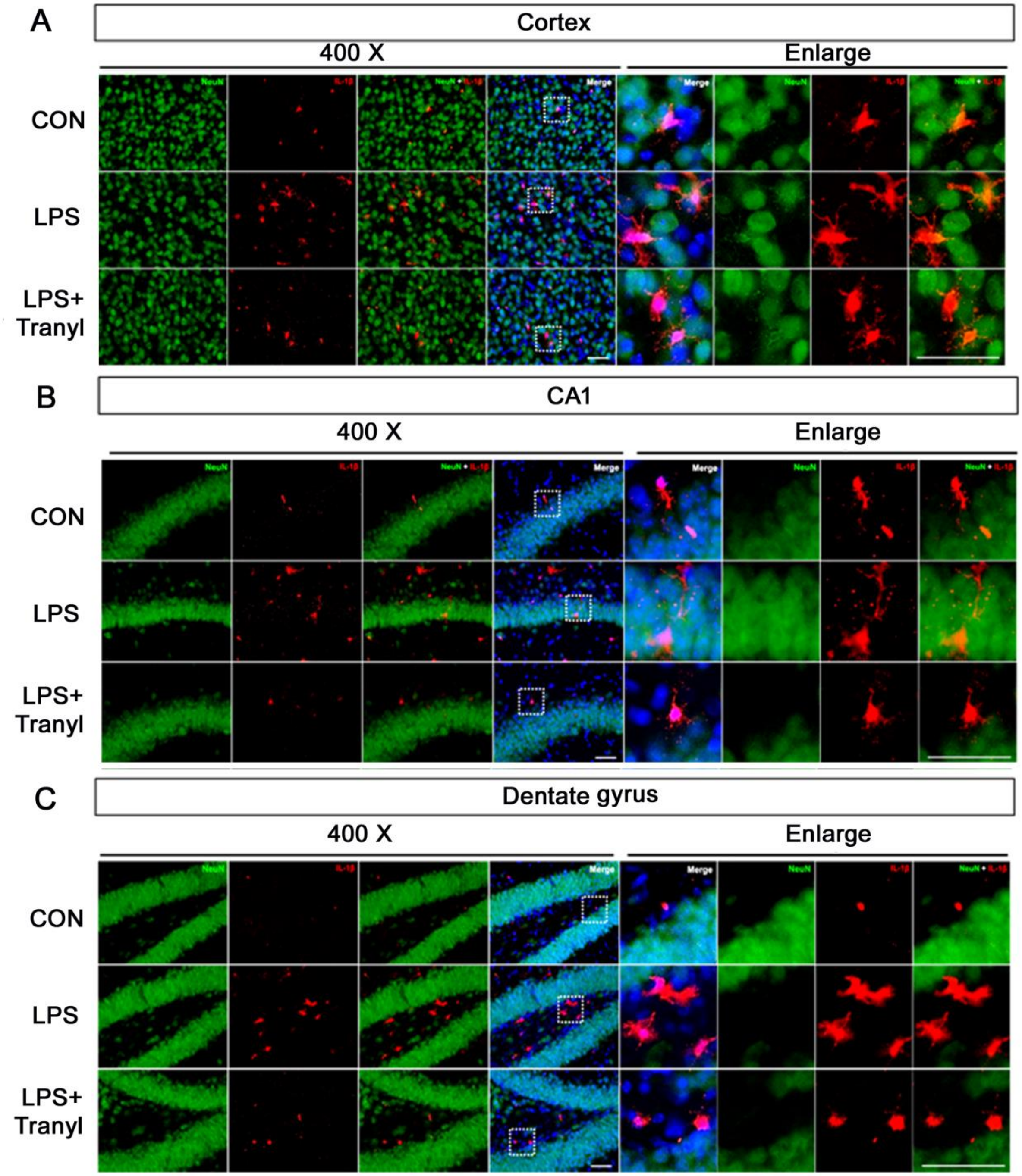

Supplementary Figure S5. The proinflammatory cytokine IL-1β does not co-localize with the neuronal cell marker NeuN in the cortex and hippocampus in LPS-injected wild-type mice. (**A-C**) After daily injection with tranylcypromine (3 mg/kg, i.p.) or PBS for 3 days, wild-type mice were injected with LPS (10 mg/kg, i.p.) or PBS. Perfused and fixed mice were then subjected to immunohistochemistry with anti-IL-1β and anti-NeuN antibodies.
